# The Effects of Different Signaling Pathways in Adenylyl Cyclase Stimulation on Red Blood Cells Deformability

**DOI:** 10.3389/fphys.2019.00923

**Published:** 2019-08-14

**Authors:** Alexey N. Semenov, Evgeny A. Shirshin, Alexei V. Muravyov, Alexander V. Priezzhev

**Affiliations:** ^1^Department of Physics, Moscow State University, Moscow, Russia; ^2^International Laser Center, Moscow State University, Moscow, Russia; ^3^Department of Medicine and Biology, Yaroslavl State Pedagogical University, Yaroslavl, Russia

**Keywords:** RBCs deformability, adenylyl cyclase, signaling pathways, laser ektacytometry, microrheology

## Abstract

Signaling pathways of red blood cells’ (RBCs) micromechanics regulation, which are responsible for maintaining microcirculation, constitute an important property of RBC physiology. Selective control over these processes may serve as an indispensable tool for correction of hemorheological disorders, which accompany a number of systemic diseases (diabetes mellitus I&II, arterial hypertension, malaria, etc.). Activation of certain pathways involving adenylyl cyclase may provide fast adaptive regulation of RBC deformability (RBC-D). However the specific molecular conditions of intracellular signal transduction in mediating RBC microrheological properties at adenylyl cyclase stimulation remain unclear. In this paper, we present the results of the *in vitro* study of the effects of different signaling pathways in adenylyl cyclase stimulation on RBC-D. We studied (1) the direct stimulation of adenylyl cyclase with forskolin; (2) non-selective adrenoreceptor stimulation with epinephrine; (3) β2-adrenoreceptor agonist metaproterenol; (4) membrane-permeable analog of cAMP (dibutyryl-cAMP). Using laser ektacytometry, we observed a concentration-dependent increase in RBC-D for all studied effectors. The EC50 values for each substance were estimated to be in the range of 1–100 μM depending on the shear stress applied to the RBC suspension. The results can serve as an evidence of adenylyl cyclase signaling cascade involvement in the regulation of RBC micromechanical properties presenting a complex molecular pathway for fast increase of microcirculation efficiency in case of corresponding physiologic metabolic demands of the organism, e.g., during stress or physical activity. Further studies of this molecular system will reveal new knowledge which may improve the quality of medical treatment of hemorheological disorders.

## Introduction

Human red blood cells (RBCs) are highly specialized cells. Their main function is to provide transport of respiration gases and various metabolic nutrients in organs and tissues. Throughout the circulatory system, RBC undergoes huge mechanical stress passing through capillaries, whose diameter is less than the mean diameter of red blood cells. Deformability of RBC (RBC-D) is a biomechanical property of erythrocytes, which allows them to reversibly change their shape and sizes to be able to squeeze through the terminal capillaries of the microvascular system. Hemorheological disorders, which accompany a number of systemic diseases such as diabetes mellitus I&II, arterial hypertension, sickle cell decease, etc. are characterized with a dramatic decrease in RBC-D, leading to the impairment of blood perfusion, which may result in irreversible tissue necrosis (Barshtein et al., 2007).

RBC-D is determined by several factors: (1) volume-to-surface ratio of RBC; (2) intracellular viscosity; and (3) structural organization of membrane and cytoskeleton (Evans and La Celle, 1975; Chien, 1977; Baskurt and Meiselman, 2003; Huisjes et al., 2018). The latter represents a system of protein–protein interactions between spectrin/actin network and integral membrane protein complexes. During evolution, the demand in maximizing the efficiency of oxygen delivery deprived RBCs of many subcellular structures such as nuclei, mitochondria, ribosomes, etc. However, RBCs preserved many elements of molecular signaling cascades which allow fast adaptive regulation of microrheological properties in response to metabolic requirements of the organism (Takakuwa et al., 1990; Muravyov et al., 2009; Muravyov and Tikhomirova, 2013; Richou et al., 2018). Triggers of such systems are various signaling molecules: catecholamines (epinephrine and norepinephrine), adrenoreceptor agonists (adrenomimetics), and hormones; effectors of ion channels and pumps. Reactive oxygen species, carbon monoxide (CO), hydrogen sulfide (H2S), and regional variations of cellular ATP can all serve as signaling triggers despite they do not have mechanisms of the specific recognition (Bogdanova et al., 2009; Diederich et al., 2018). Their action alters the transport potential of hemoglobin, which in this particular case acts as a biosensor (Luneva et al., 2015). Nitric oxide (NO) as a signaling molecule regulates many processes affecting hemorheology (Bor-Kucukatay et al., 2003; Chen and Popel, 2009; Ulker et al., 2011). The exposure of RBC to donors of NO led to the positive changes of RBC microrheological properties (Mozar et al., 2016). Experiments demonstrated that at hypoxic vasodilation, erythrocytes act as a NO generator upon activation of RBC NO-synthase (NOS) (Grau et al., 2013). That improves local microrheology contributing to vascular smooth muscle relaxation (Baskurt et al., 2004). One of the key questions is whereas both oxygen (O_2_) and NO diffuse into RBC, only O_2_ can diffuse out (Pawloski et al., 2001). The data on the mechanisms of NO export from RBC into the blood stream as well as its effects on RBC microrheology are very contradictory and being very intensively studied (Singel and Stamler, 2004; Ulker et al., 2018; Sun et al., 2019).

Adenylyl cyclase (AC) signaling cascade is of a particular interest for studies of the mechanisms of RBC-D adaptive regulation (Sprague et al., 2005; Muravyov et al., 2010). Its core element is an enzyme adenylyl cyclase which synthesizes the cyclic form of AMP (cAMP) from ATP upon stimulation of the G-protein-coupled receptor (GPCR). The AC-cascade can be triggered by the signaling molecules such as prostaglandins, prostacyclins, hormones and agonists of adrenoreceptors. That is why the AC-cascade is called a “stress”-signaling cascade. Six distinct classes of AC have been described, all catalyzing the same reaction but representing unrelated gene families. ACs class III are of a great interest due to their important role in human health. In this class there are nine isoforms of membrane ACs divided into four groups (types) according to the regulatory properties (Sadana and Dessauer, 2009). The existing data indicate that mammalian RBCs possess the adenylyl cyclase type II, which is activated by the heterotrimeric GTP-binding proteins (G-proteins) (Sprague et al., 2005). AC-cascade is presented as a typical system of intercellular signaling in many cells, including RBC (Kaiser et al., 1974). There is a strong evidence of its functioning in RBC being responsible for the adjustments of the micromechanical properties of the cell (Thomas et al., 1979; Oonishi et al., 1997; Horga et al., 2000).

**Figure 1:**
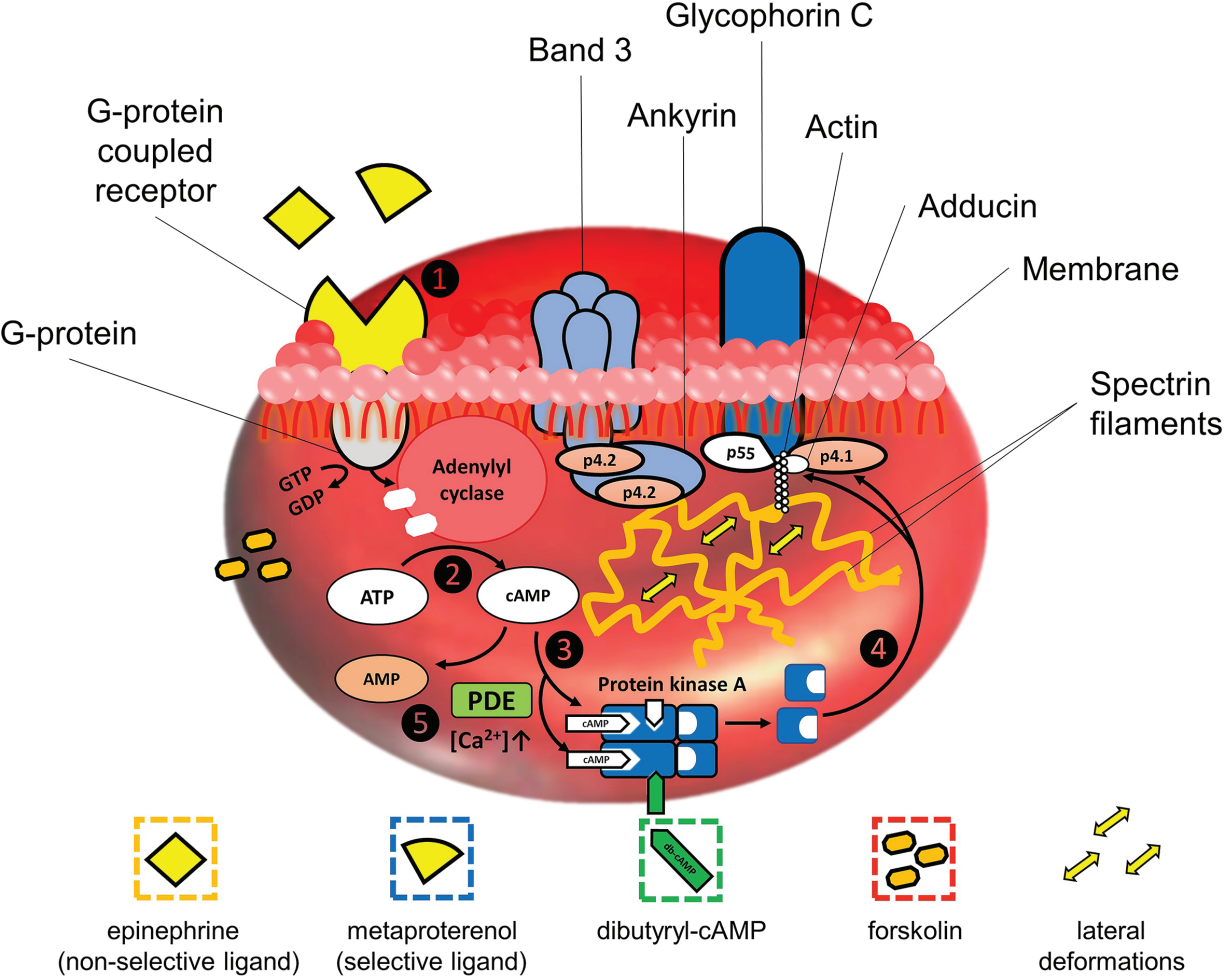
Simplified stepwise block-scheme of signaling pathways in adenylyl cyclase stimulations: step 1 – bounding of a ligand by G-protein-coupled receptor on RBC membrane leads to the activation of a G-protein, which hydrolyzes GTP to GDP; step 2 – released energy is consumed for the activation of adenylyl cyclase, which starts synthesis of cAMP from ATP; step 3 – cAMP molecules bind to each of the regulatory subunits, and the bonds between regulatory and active units of cAMP-dependent protein kinase A (PKA) are disintegrated, converting it to the activated form; step 4 – catalytic centers of PKA now are able to phosphorylate serine residues of p4.1 in junction complexes of RBC cytoskeleton causing the local disintegration of spectrin network, subsequently resulting in RBC deformability increase; step 5 – functioning of negative feedback mechanism involving calcium-sensitive forms of phosphodiesterases (PDE), whose activation upon Ca^2+^-influx leads to the decrease of cAMP due to its hydrolysis to monophosphate form.

The simplified scheme of AC-cascade is demonstrated in Figure 1. Binding of a ligand by the GPCR on the RBC membrane leads to the activation of a G-protein, which hydrolyzes GTP to GDP upon activation (step 1 in Figure 1). The released energy is consumed for the activation of membrane AC which starts the synthesis of cAMP from ATP (step 2). An increase in cAMP concentration serves as a stimulus for the cAMP-dependent protein kinase A (PKA) activation. PKA is a heterodimer comprising four subunits – two catalytic and two regulator units (Rubin, 1979). Being inactivated, the catalytic (enzymatic active) centers are blocked by the regulator segments. As the concentration of cAMP increases, two cAMP molecules bind to each of the regulatory subunits (Berg et al., 2011). The bonds between the regulatory and active units are disintegrated and PKA converts to the activated form (step 3). Catalytic centers of PKA are released and become able to phosphorylate the serine residues of proteins in junction complexes of the RBC cytoskeleton (step 4), subsequently resulting in the cell deformability increase.

Phosphorylation/dephosphorylation (attachment/removal of the phosphate group) of membrane protein residues leads to the conformational changes of protein molecular structure (Boivin et al., 1981). Phosphorylation of the membrane proteins residues by protein kinases causes degradation of the corresponding complexes providing an increase in RBC plasticity and elasticity. Phosphorylation of p4.1 by PKA *via* serine residues (Ling et al., 1988; Gauthier et al., 2011) or by members of the protein kinase C (PKC) family *via* hydroxyl groups of serine and threonine residues (Palfrey and Waseem, 1985) perturbs the bonds of the whole complex with spectrin, leading to the degradation of the junction causing the disintegration of the network. This process is believed to be responsible for lateral (horizontal) deformations of RBCs. Stimulated by cAMP analogs, phosphorylation of adducin also may play a role in mediating the interactions of components of the cytoskeleton with the inner surface of the membrane in RBC (Waseem and Palfrey, 1988). Phosphorylation of p4.2 leads to the degradation of ankyrin complex but does not disintegrate the spectrin network. It leads to the detachment of the spectrin network from the membrane allowing vertical deformations (Kaushansky et al., 2016). Residues of p4.2 are possible targets for PKA. However, based on the rates of disintegration of band 3-ankyrin linkages, it was concluded that the kinetics of the band 3-ankyrin intermolecular interaction is too slow for the adaptive microrheological reaction (Anong et al., 2006).

AC-cascade cannot function without negative feedback; otherwise, adenylyl cyclase would consume all ATP in the cell. The role of a negative feedback agent is played by phosphodiesterases (PDE) – a wide class of enzymes capable of hydrolyzing the phosphodiester bonds (Adderley et al., 2009). The activity of PDE leads to a decrease in cAMP due to its hydrolysis to the monophosphate form (step 5 in Figure 1).

The aim of the present work is to investigate the concept, which supports the idea that activation of the AC-cascade physiologically occurs in RBC and leads to the fast adaptive increase in deformability capable of improving microcirculation efficiency at stress or high physical duties. We suggest the necessity to investigate the effects of different signaling pathways in adenylyl cyclase stimulation on red blood cells’ deformability, including GPCR stimulation, direct activation of adenylyl cyclase, and cAMP-dependent PKA stimulation. For that purpose, we studied the effects of (1) non-selective adrenoreceptor stimulation with epinephrine (E4250, Sigma Aldrich); (2) selective β2-adrenoreceptor stimulation with metaproterenol (M2398, Sigma Aldrich); (3) the direct stimulation of adenylyl cyclase with forskolin (F6886, Sigma Aldrich); (4) membrane-permeable analog of cAMP (dibutyryl-cAMP, db-cAMP, D0627, Sigma Aldrich).

## Materials and Methods

### Red Blood Cell Suspension Preparation and Composition of Experimental Solutions

RBCs were extracted from 10 healthy male donors 20–30 years of age by gentle finger prick method using a sterile lancet. This method was used due to several reasons: (1) when sampling from the vein, the resin cord blocks the blood flow, leading to the uncontrolled increase of the blood pressure. That disturbs the local microrheological profile due to the release of the signal molecules, affecting RBC. All of these factors lead to difficulties at experimental data interpretation; (2) hematocrit (Hct) values may significantly differ when blood is taken from the vein. In the protocol of the present study, the capillary blood was taken without pressuring, which allowed to contain more stable Hct values; (3) the volume of the blood, required for each RBC-D ektacytometry measurement, is 10 μl, therefore blood sampling from the vein is not rational.

After the finger pricking, 10 μl of the donor’s blood was collected into isotonic PBS (phosphate buffered saline, pH 7.4, Gibco). The present experimental protocol did not include usage of the blood anticoagulant: we briefly checked the effect of forskolin on RBC-D at different types of the blood stabilization (the results are presented in Supplementary Material Section 9). The increase in RBC-D at forskolin (10 μM) was observed regardless of anticoagulant presence. Right after the extraction, RBCs were washed in isotonic PBS three times (for 3 min, 2,500 g, room temperature). After the third wash, the supernatant was collected and changed with the experimental solution, preliminary warmed to 37°C, for further incubation.

Stock solutions of the substances were obtained by the dilution of dry samples in PBS [epinephrine (100 mM), metaproterenol (100 mM), db-cAMP (5.2 mM)], and DMSO [forskolin (6 mM)]. Experimental solutions for cells’ incubation were obtained by sequential dilutions of aliquots of stock solutions in PBS. As adrenergic receptors activate adenylyl cyclase in human erythrocyte membranes at physiological calcium plasma concentrations (Horga et al., 2000), all experimental solutions were supplemented with calcium chloride (CaCl2, C1016, Sigma) so that the final concentration of calcium was 1 mM. Washed RBCs were incubated in 1% suspension in aliquots of the studied substances for 15 min at 37°C. After incubation, the cells were washed once, supernatant was collected, and the cells were re-suspended in 0.14 mM polyvinylpyrrolidone (PVP360, Sigma Aldrich; viscosity of 30 cP at 37°C; pH = 7.4; osmolarity: 290 mOsm/l) at 1% Hct. pH of the PVP gel was measured every time in each set of the measurements using indicator test strips (P4536, Sigma Aldrich). All parameters correspond with the values recommended by laser ektacytometer RheoScan-D manufacturer.

All methods and experiments were carried out in accordance with recommendations for hemorheological laboratories developed by the international expert group created for hemorheological research standardization (Baskurt et al., 2009a,b). The study was approved by the Local ethics committee of the Medical Scientific and Educational Center of M. V. Lomonosov Moscow State University (Permission document № 1/19). All participants gave written informed consent in accordance with the Declaration of Helsinki.

### RBC-D Measurements and Analysis

RBC-D was measured using the ektacytometer RheoScan-D (RheoMediTech, Korea) which utilizes the laser diffractometry technique. In this method, measurements of RBC-D are made by analyzing the changes of diffraction patterns, obtained by illuminating the RBC suspension by the laser diode (635 nm, 1.5 mW), due to shear stresses occurring in a flow (Groner et al., 1980). When external mechanical stresses are low, the RBCs are not deformed but oriented so that the shape of their cross-section is close to a circle and the corresponding diffraction pattern from such a sample is circularly shaped. Under high shear stresses, the RBCs are elongated, taking shapes close to ellipsoidal and so does the average diffraction pattern, which is a result of the superposition of thousands of diffraction patterns from individual cells. Analyzing the degree of ellipsoid elongation of diffraction pattern allows for measuring the average deformability of the RBC population as a function of shear stress in the range from 0.5 to 20 Pa in terms of elongation index (EI) (Groner et al., 1980; Shin et al., 2005; Nikitin et al., 2016). The dependence of EI on shear stress is called the deformability curve. The detailed description of RheoScan-D principles is available in the publications of its developer’s group (Shin et al., 2005, 2007) and a brief description is presented in Supplementary Material Section 1.

The EI values of the RBC suspension for each stimulator concentration were analyzed for a wide range of shear stresses (SSs). In order to assess peculiarities of adenylyl cyclase stimulation effects on RBC-D, we measured EI dependence on SS in the whole range of shear stresses from 0.1 to 20 Pa. We were able to analyze the behavior of EI at various concentrations of AC-cascade stimulators and assess EC50 values at different SSs. The latter was performed by fitting the dependence of EI on the concentration of the stimulators using sigmoidal approximation formula:

EIx=A1−A21+xEC50a+A2

where *a* is a parameter, *x* is the concentration of AC stimulator, *A_1_* and *A_2_* are the highest and the lowest observed values, and EC50 is the half-maximal effective concentration. The fitting was performed in software package Origin 2018. The graph of the function is presented in Supplementary Material Section 3. Standard parameters EImax and SS1/2, described in (Baskurt et al., 2009a,b), were also estimated. The description of the data approximation formula implemented for each AC-cascade stimulator to obtain EImax and SS1/2 values is available in the Supplementary Material Section 4.

### Statistics and Data Presentation

Experimental data were analyzed in software package Statistica 12. The number of measurements (repeats) at every concentration was not less than 10 for each donor. Statistical significance value of *p* was estimated using standard *t* test for independent variables.

## Results

**Figure 2:**
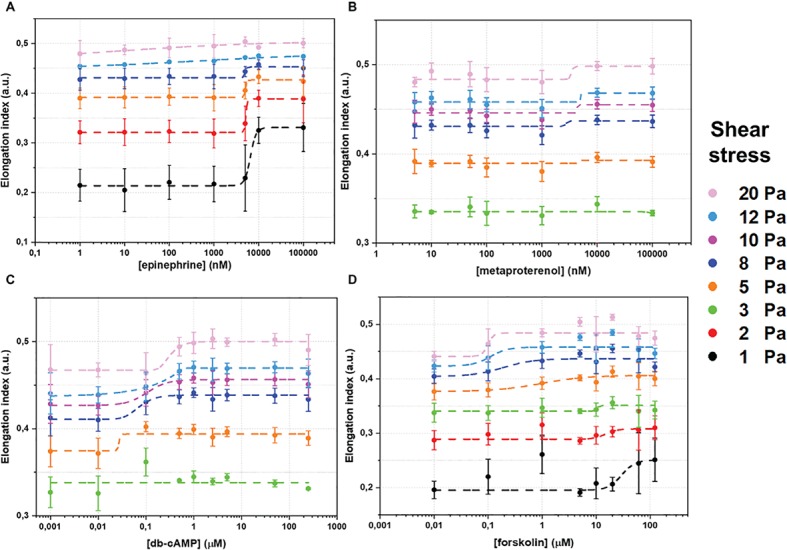
Changes of red blood cells deformability (RBC-D) in dependence on concentration of adenylyl cyclase signaling cascade stimulation agents: **(A)** – epinephrine; **(B)** – metaproterenol; **(C)** – dibutyryl-cAMP (db-cAMP); **(D)** – forskolin. RBC-D was assessed by ektacytometry. Change in laser diffraction pattern was measured by recording the signal designated as elongation index (EI) as a function of shear stress. RBCs were incubated in experimental solutions at 1% Hct at 37°C for 15 minutes. Number of donors *N* = 10, error bars represent standard deviations from mean values. Dashed curves represent fitting using sigmoidal function. The goodness of fitting *r*^2^ was not less than 0.97–0.99.

**Figure 3:**
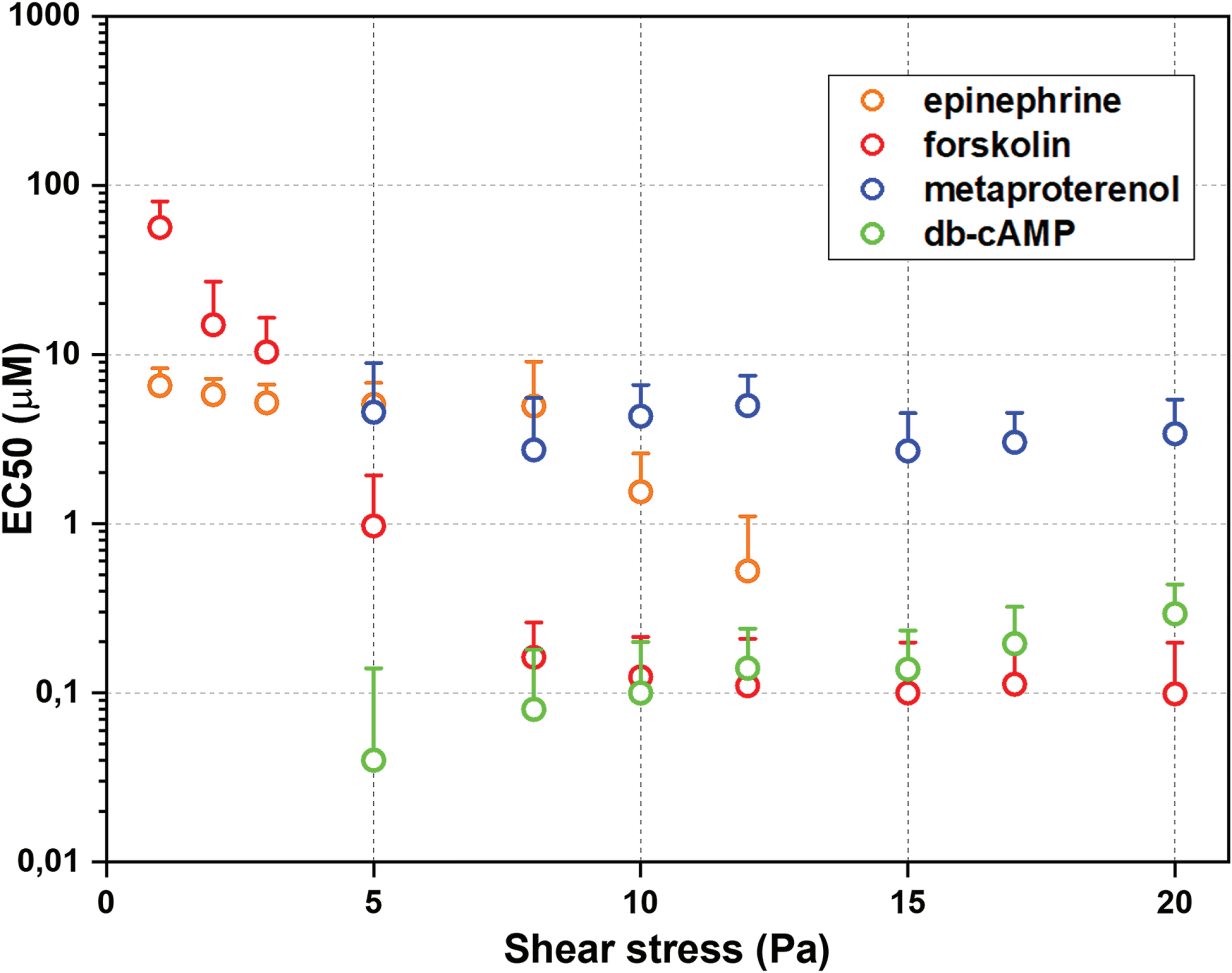
Half-maximal effective concentrations (EC50) estimated using laser ektacytometry data. Error bars indicate confidence intervals obtained at the best parameters of fitting ektacytometry data with sigmoidal function.

The whole data set of measured RBC-D curves is presented in the Supplementary Material Section 2. Figure 2 demonstrates sigmoidal curves of EI dependences on concentrations of different stimulators of AC signaling cascade at different shear stresses. Effects of epinephrine are demonstrated in Figure 2A. It is seen that the presence of epinephrine (Figure 2A) in concentration up to 1 μM does not change RBC-D under all shear stresses. Starting from 1 μM, we observed an increase in EI reaching saturation. Such behavior was observed for low and medium shear stresses in the range of 1–8 Pa. Applying high shear stresses (>10 Pa) led to a linear increase in EI and saturation was not observed. Thus, the epinephrine dependence of half-maximal effective concentration (EC50) values on shear stress was observed (Figure 3, orange circles): EC50 was 6.5 ± 1.7 μM at 8 Pa and dropped to 1.6 ± 0.5 μM below 1 Pa and could not be calculated at shear stresses over 10 Pa.

The dependence of EI on concentration of metaproterenol was different from that for epinephrine (Figure 2B). At shear stresses below 3 Pa, metaproterenol did not influence EI, but starting from 5 Pa, an increase in EI appeared and became more significant with the growth of the shear stress. In case of metaproterenol, EC50 (where it was possible to calculate) did not significantly depend on shear stress and was in the range 2.7 ± 0.8 to 4.5 ± 4.2 μM (Figure 3, blue circles). The effect of db-cAMP was similar (Figure 2C): there was no effect at shear stresses below 3 Pa but EI started to increase when shear stresses became higher (over 5 Pa). In the range 5–20 Pa, the EC50 values were 0.1–0.3 μM demonstrating a small linear increase with shear stress (Figure 3, green circles).

The direct stimulation of adenylyl cyclase with forskolin increased EI at every shear stress starting from 1 Pa (Figure 2D). However, the character of its influence depended on the shear stress. This phenomenon is represented in the dependence of forskolin EC50 values on shear stress (Figure 3, red circles): at low shear stresses below 3 Pa, EC50 was high reaching 56 ± 24 μM but dramatically dropped down to 1 μM at medium shear stress (around 5 Pa) and further to 0.1 μM at high stress (>8 Pa).

The values of EImax and SS1/2 parameters are available in the Supplementary Material Sections 5 and 6. The dependences of EImax on the concentration for each AC-cascade stimulating agent was approximated with sigmoidal function: the obtained EC50 values coincide by the order of magnitude with values obtained from analysis of the deformability curves. The behavior of SS1/2 parameter was different: in case of epinephrine and forskolin, SS1/2 trend correlates with the data obtained from deformability curves, while for metaproterenol and db-cAMP, the significant changes of SS1/2 were not observed. That can be explained by the threshold in the shear stress to observe the effect of db-cAMP and metaproterenol on RBC-D.

## Discussion

**Figure 4:**
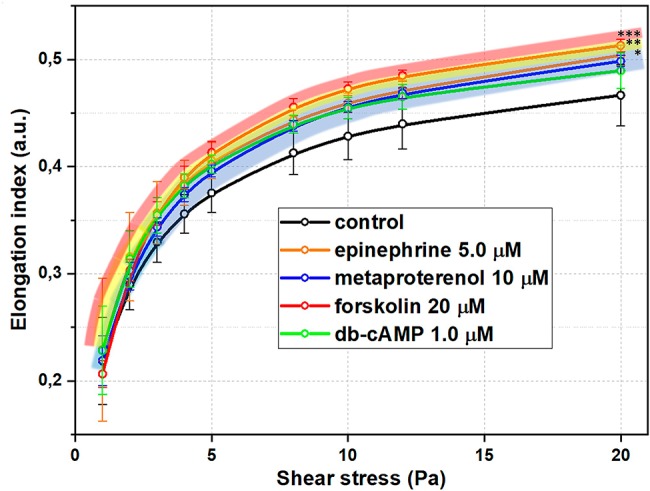
Red blood cells deformability (RBC-D) changes upon stimulation of adenylyl cyclase signaling cascade at concentrations close to EC50 values for each substance. RBC-D was assessed by ektacytometry. Change in laser diffraction pattern was measured by recording the signal designated as elongation index (EI) as a function of shear stress for each concentration. RBCs were incubated in experimental solutions at 1% Hct at 37°C for 15 minutes. Control samples are intact cells incubated under the same conditions in PBS. Number of donors *N* = 10, error bars represent standard deviations from mean values. Presented significances are relative to control values, *p* was estimated using standard *t* test for independent variables (^*^p < 0.05, ^**^p < 0.01, ^***^p < 0.001).

Summarized effects of different signaling pathways in AC stimulation on RBC-D are demonstrated in Figure 4 at concentrations of substances around corresponding EC50 values. RBC deformability curves for each studied substance in a wide range of concentrations are presented in Supplementary Material Section 2. In our pilot study (Semenov et al., 2018), we demonstrated the principal sensitivity of laser ektacytometry technique in detecting the increase in RBC-D upon stimulation of adenylyl cyclase. Here we studied this phenomenon in a more complex and detailed way.

Firstly, in all cases of AC-cascade stimulation, we observed a concentration-dependent increase in RBC-D without morphology alterations of RBC even at the highest concentrations of the substances. In Supplementary Material Section 7, we present the microphotographs of the blood smears containing the experimental RBC suspension in PVP solution under two glass slides at the highest concentrations of adenylyl cyclase cascade stimulators (epinephrine 100 μM, metaproterenol 100 μM, db-cAMP 50 μM, forskolin 60 μM). We can see that there are no alterations of cellular shape in comparison with intact RBC sample: cells preserve their physiological discoid shape. Secondly, the effects of AC-cascade stimulation were found to be complex as in some particular cases they were dependent on the shear stress.

Effects of epinephrine and especially forskolin demonstrated the most dramatic dependence on shear stress: EC50 decreased when shear stresses were getting higher. EC50 for metaproterenol and db-cAMP did not change much with the shear stress, but in their cases, there was a threshold in shear stress around 3 Pa, below which significant changes in RBC-D were not observed.

Forskolin effect dependence on the shear stress can be explained by its wide non-selectivity and complexity. Forskolin is capable of direct activation of adenylyl cyclase resulting in the increase in intracellular cAMP concentration (Seamon et al., 1981). It was demonstrated (Wood et al., 2006) that forskolin enhanced phosphorylation of α-Ser485/491 by the cAMP-dependent protein kinase which may play a role in changes in RBC-D. Indeed, in Muravyov et al. (2009), it was demonstrated that the shear-induced elongation of RBC pre-incubated with forskolin (10 μM) was significantly higher. Forskolin also was found to be a potent inhibitor of glucose transport in human erythrocytes: the inhibitory effect was instantaneous, reversible, and concentration-dependent, having an IC50 value of 7.5 μM (Sergeant and Kim, 1985). The biochemical aspects of glucose transport’s influence on RBC-D are not completely understood and are being widely investigated nowadays (Galiniak et al., 2015). Also, the final effects of cAMP production are as diverse as the cells that respond to forskolin. So far, the effects of forskolin depend on the adenylyl cyclase isoforms expressed in each kind of cell (Pinto et al., 2008; Huerta et al., 2014).

Examples of GPCR on RBC membrane are the adrenergic receptors. There are data on the existence of β1- and β2-types of adrenoreceptors on RBCs membrane (Bree et al., 1984) and β2-subtype is predominant as it is expressed two times higher. Several studies suggest that the micromechanics of RBC can be modulated by adrenomimetics (adrenoreceptors agonists) while the developments of the effects are controversial. In an early work (Allen and Rasmussen, 1971), it was suggested that human RBCs respond to epinephrine and β2-adrenomimetic isoproterenol with a decrease of filterability and the epinephrine dose–response curve was biphasic. However, later on, more studies reported the opposite. In Chiocchia and Motais (1989), the results showed that stimulation by catecholamines increased the erythrocyte deformability. In work by Tuvia et al. (1999), it was demonstrated that exposure of RBC to adrenaline elevated red cells’ membrane fluctuation and filterability. The authors explained the effects by transducing mechanisms *via* cAMP-dependent pathway. Their conclusions are supported by the early work of Tsukamoto and Sonenberg (1979) where it was shown that adrenergic agonists (−)isoproterenol (2 μM) and (−)epinephrine (10 μM) stimulated the cAMP-dependent PKA in RBC. The effects of epinephrine on RBC-D were studied during low-intensity irradiation with He-Ne laser at power output 8.0 mW and 632.8 nm wavelength (Yova and Koutsouris, 1994): the authors reported on the increase in RBC-D in the presence of epinephrine at concentration 100-1000 µM. In a study by Hilario et al. (2003) involving 42 male and female donors, the authors aimed at *in vitro* verification of the effect of adrenaline (10 μM) and it was shown that deformability of peripheral blood erythrocytes was non-significantly increased in both sexes while the effect on membrane fluidity was different: adrenaline decreased male and increased female values. Attempts of using optical techniques (interferometry, UV, FTIR, and IR spectroscopy) made in Kunitsyn and Panin (2013) on erythrocyte ghosts suggested that under the influence of adrenaline the α-helix→β-structure transition occurs to adrenoreceptor leading to the further structural mechanical change of the membrane. Metaproterenol is a selective β2-adrenomimetic which is used in drug treatment of asthma, bronchitis, and emphysema (Bosak et al., 2012). Its influence on RBC-D was more stable in accordance with shear stress as β2-adrenergic receptors are associated with stimulation of Gs-protein/adenylyl cyclase activity (Muravyov and Tikhomirova, 2013). Epinephrine presents non-selective adrenoreceptor stimulation, affecting α- and β-subtypes. Different subtypes of adrenergic receptors are coupled with different intracellular signal transduction mechanisms (Muravyov et al., 2009), and thus their stimulations act differently in RBC micromechanical regulation. That can explain the observed shear stress dependence of the effects of epinephrine.

Increase in RBC-D was observed for dibutyryl-cAMP (db-cAMP) – cell-permeable analog of cAMP that activates the cAMP-dependent PKA. In human erythrocytes, db-cAMP induces the phosphorylation of p4.1 on sites within the adjacent 16 kDa and 10 kDa chymotryptic domains (Horne et al., 1990) and the 10-kDa segment contains the spectrin/actin-binding site (Correas et al., 1986). Influence of db-cAMP is partially selective, and thus it demonstrated a non-significant dependence on the shear stress. To support our findings about the role of AC-cascade stimulation on the single-cell level, we performed an experiment involving elongation of the single RBC using optical (laser) tweezers in the presence of epinephrine and db-cAMP. The methodology and the results are presented in Supplementary Material Section 10. We observed that in the presence of epinephrine (10 µm) or db-cAMP (1 µm) in the experimental solution, the RBCs were elongated easier in comparison with the intact RBCs. The effect of epinephrine was more significant, which corresponds with the laser ektacytometry results. In both cases, the elongation was non-linear in dependence on the external mechanical stimuli.

The role of the external mechanical stimuli in the changes of biochemical conditions of RBC is one of the challenging problems in the study of the mechanisms of active RBC-D regulation (Simmonds et al., 2014). The question how biomechanical response of the RBC changes according to the external stress applied to the cell is very important for the designing artificial cardiovascular devices supporting blood circulation, in which the external shear stress (SS) may reach an extremely high level > 50 Pa (Lee et al., 2004). At such stresses, RBCs are close to hemolytic affection and exposed to mechanical trauma.

Exposition of RBCs to SS in the physiologically normal range 5–20 Pa, on the contrary, may reversibly improve the deformability (Simmonds et al., 2014; Nemeth et al., 2018). The exact mechanisms of such phenomena still remain unclear. In Meram et al. (2013), the authors indicate that systems of intracellular signal transduction including receptors, intermediates, and final targets (phosphorylation/dephosphorylation of protein residues) can be involved. This phenomenon can be the mechanism underlying the observed shift of EC50 values of AC-cascade stimulators in SS range 1–20 Pa. We suggest that the timing of RBC’s response to the selective AC-cascade stimulation with metaproterenol is fast enough while less-selective activation with epinephrine and forskolin governs a certain delay, at which the effect of additional SS-induced increase in RBC-D may occur. The biophysical interpretation is complicated with “cross-talks” of signaling systems in RBC, especially between AC-cascade and calcium signaling. Alterations of Ca^2+^-influx in RBC under application of shear stress are reported in several works and according to the recent data, this process reflects the response to mechanosensitive Piezo1 receptor stimulation (Cahalan et al., 2015; Kuck et al., 2018). This effect may be responsible for the EC50 shift in case of db-cAMP as it stimulates overall cAMP-dependent protein kinase activity in the cell, which is highly sensitive to cytosolic calcium (Rasmussen and Barrett, 1984).

In recent years, the importance of the studies of signaling pathways involving adenylyl cyclase in RBC micromechanics regulation has become more and more apparent. Adenylyl cyclase cascade represents physiological opportunities to maintain and control RBCs microrheology on cellular level supporting the role of red cells as sensors and regulators of the blood flow in microcirculation and tissue perfusion. Contradictory data on adenylyl cyclase functioning in RBC (Pfeffer and Swislocki, 1976; Piau et al., 1978) are explained by its relatively low activity (Sager and Jacobsen, 1985) and complexity of biophysical organization of the cascade. The use of laser ektacytometry allowed for detection of the dose-dependent increase in RBC-D at different pathways upon AC-cascade stimulation with epinephrine, metaproterenol, db-cAMP, and forskolin. This can serve as an evidence of the involvement of AC-cascade in mechanisms of RBC-D regulation. To support the specificity of the obtained results, we performed a brief ektacytometry experiment of the effects of bisoprolol fumarate on RBC-D (the results are presented in the Supplementary Material Section 8). Bisoprolol is a selective β1-adrenoreceptor antagonist capable of blocking cAMP synthesis pathway (Galandrin and Bouvier, 2006). We observed that upon epinephrine-induced stimulation of the AC-cascade, there is an inhibitory effect of bisoprolol on RBC-D. Estimation of the EC50 values for each AC stimulator at various shear stresses, which was performed in the current study, was done for the first time. It revealed novel data about changes of functioning of RBC intracellular signaling cascades at different mechanical stresses. That phenomenon may underlie mechanisms of adaptive regulation of RBC microrheology and require further investigations.

## Data Availability

All datasets generated for this study are included in the manuscript and/or the Supplementary Files.

## Ethics Statement

All methods and experiments were carried out in accordance with recommendations for hemorheological laboratories developed by the international expert group created for hemorheological research standardization (Baskurt et al., 2009a,b). The study was approved by the Local ethics committee of the Medical Scientific and Educational Center of M.V. Lomonosov Moscow State University (Permission document № 1/19). All participants gave written informed consent in accordance with the Declaration of Helsinki.

## Author Contributions

AS conducted the experiments, developed experimental protocols, organized the materials supply and collection of the donors blood, prepared figures and graphs, and wrote the first version of the manuscript. ES suggested the algorithms of RBC deformability data analysis and conducted the general nerve of the work. AM controlled the correctness of the experimental protocols and encouraged AS to investigate signaling cascades in RBC physiology. AP encouraged AS on the laser-optics study of signaling systems functioning in the living cells and controlled the quality of laser ektacytometry measurements. All authors participated in the editing and preparation of the final manuscript and agree to be accountable for the content of the work.

### Conflict of Interest Statement

The authors declare that the research was conducted in the absence of any commercial or financial relationships that could be construed as a potential conflict of interest.
