# Supplementary material for: The Effects of Different Signaling Pathways in Adenylyl Cyclase Stimulation on Red Blood Cells Deformability
Source: Front Physiol. 2019 Aug 14;10:923. doi: 10.3389/fphys.2019.00923 (PMC6702543; doi:10.3389/fphys.2019.00923)
Supplement: Supplementary file 1 [file Data_Sheet_1.pdf]

1. Laser ektacytometry.
RheoScan-D operation scheme.

$$RBC\ Elongation\ \ index, EI = \frac{A - B}{A + B}$$

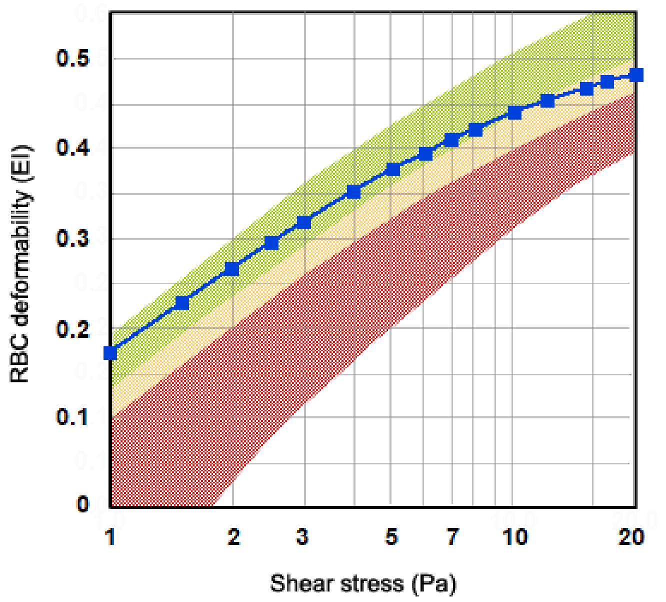

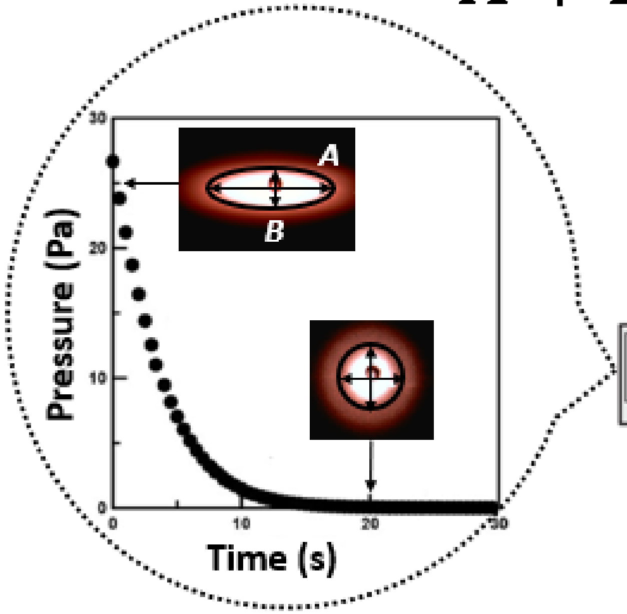

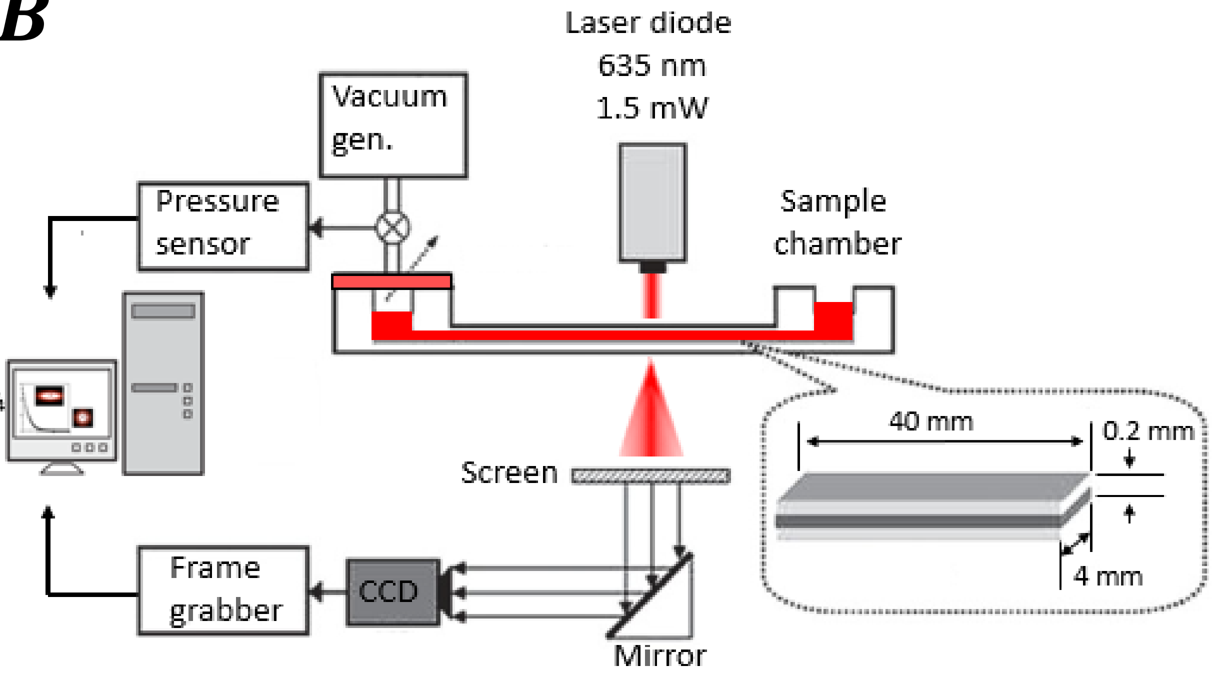

Shin S., et al. Slit-flow ektacytometry: laser diffraction in a slit rheometer // Cytometry Part B, 65B, pp. 6-13 (2005)

2. RBC deformability curves under adenylyl cyclase cascade stimulation.

The whole data set of RBC-D curves measured at different stimulation of adenylyl cyclase cascade using RheoScan-D. Number of donors N=10. Demonstrated the mean values; error bars show standard deviations.

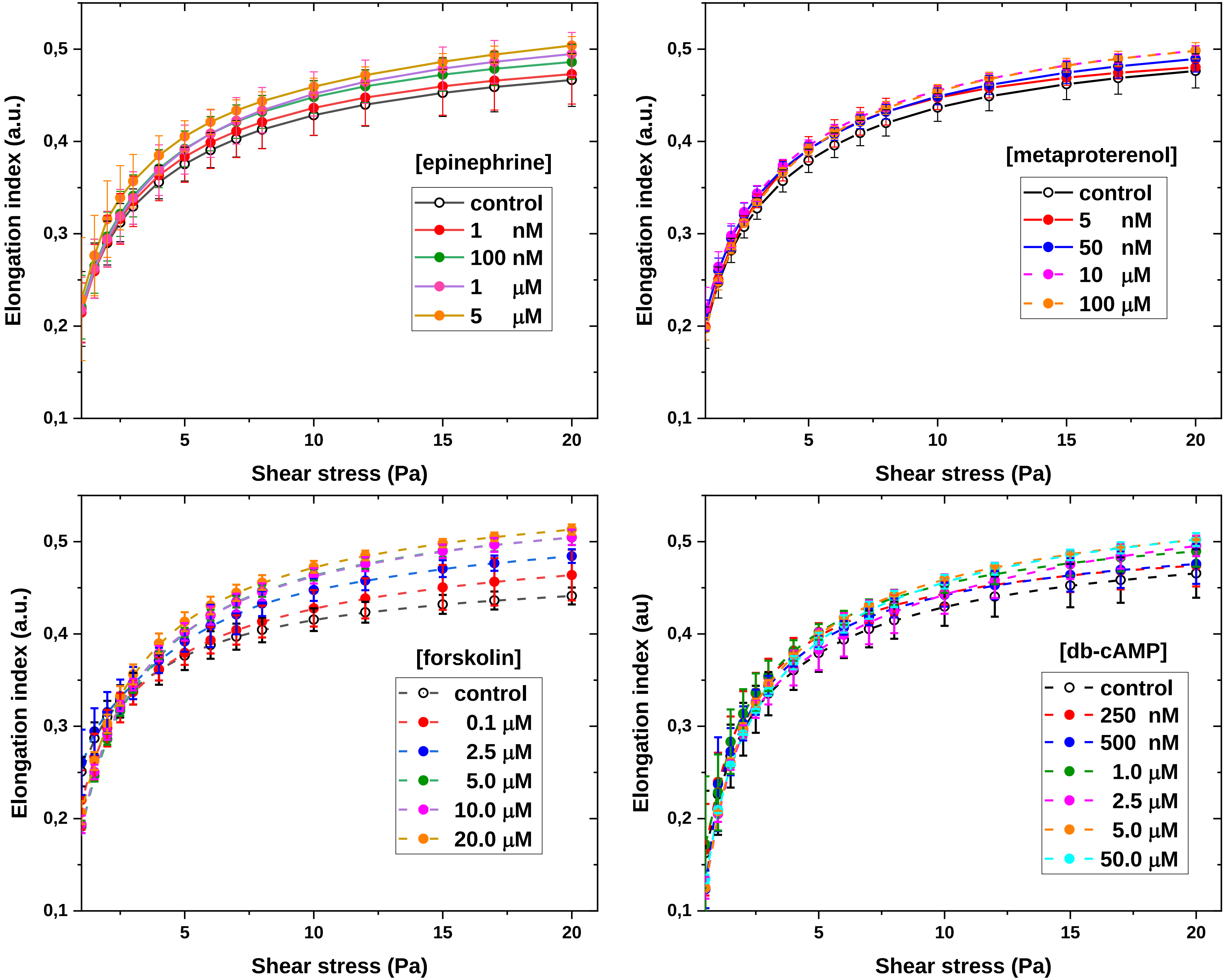

3. RBC deformability data sigmoidal approximation. EC50 values calculation.

Approximation formula

$$EI(x) = \frac{A_1 - A_2}{1 + (x/EC50)^a} + A_2$$

$$R + L \rightleftarrows RL$$

R – receptor

L – ligand (cascade stimulation agent)

RL – receptor-ligand complex

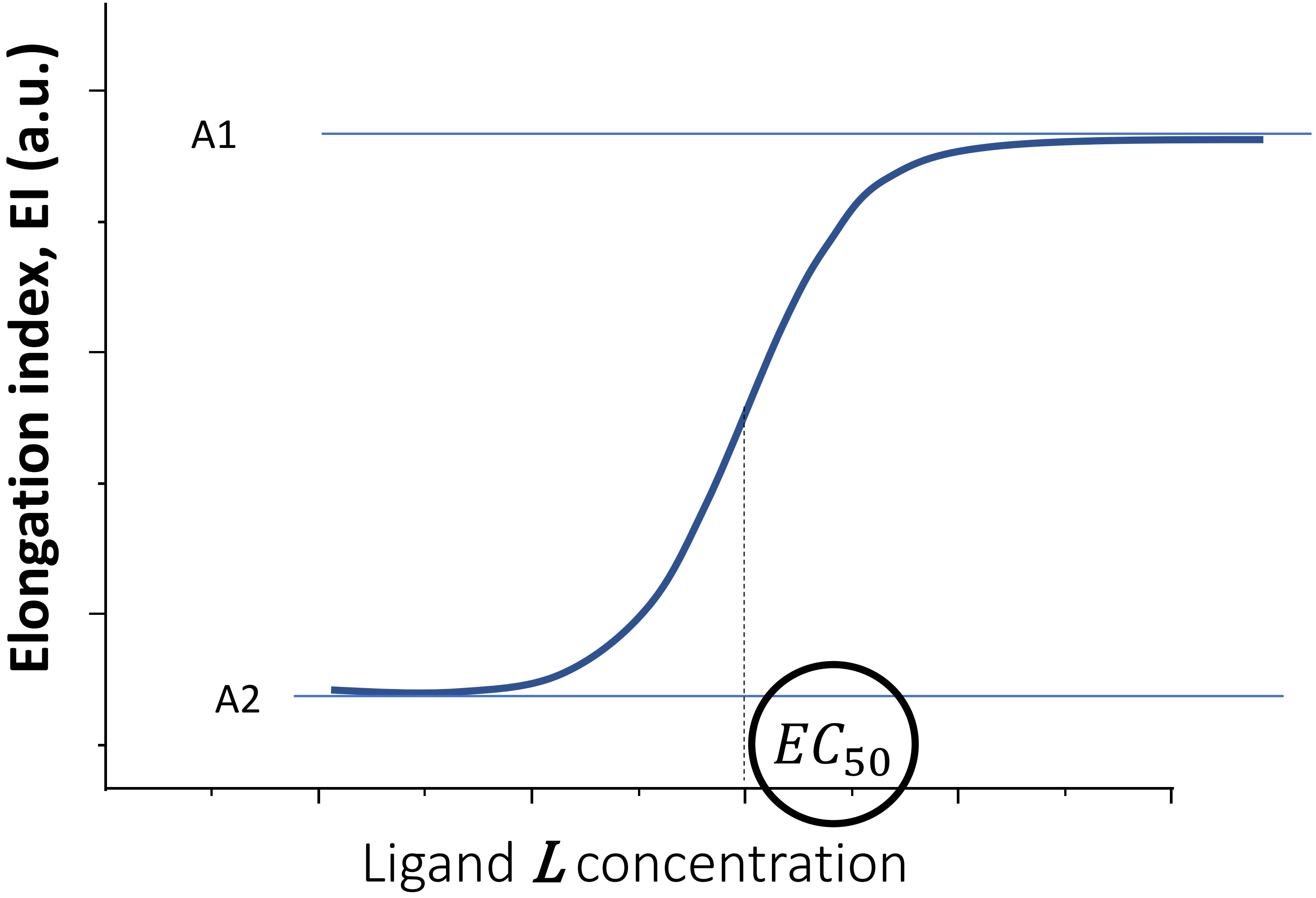

4. RBC deformability data approximation. EImax & SS1/2.

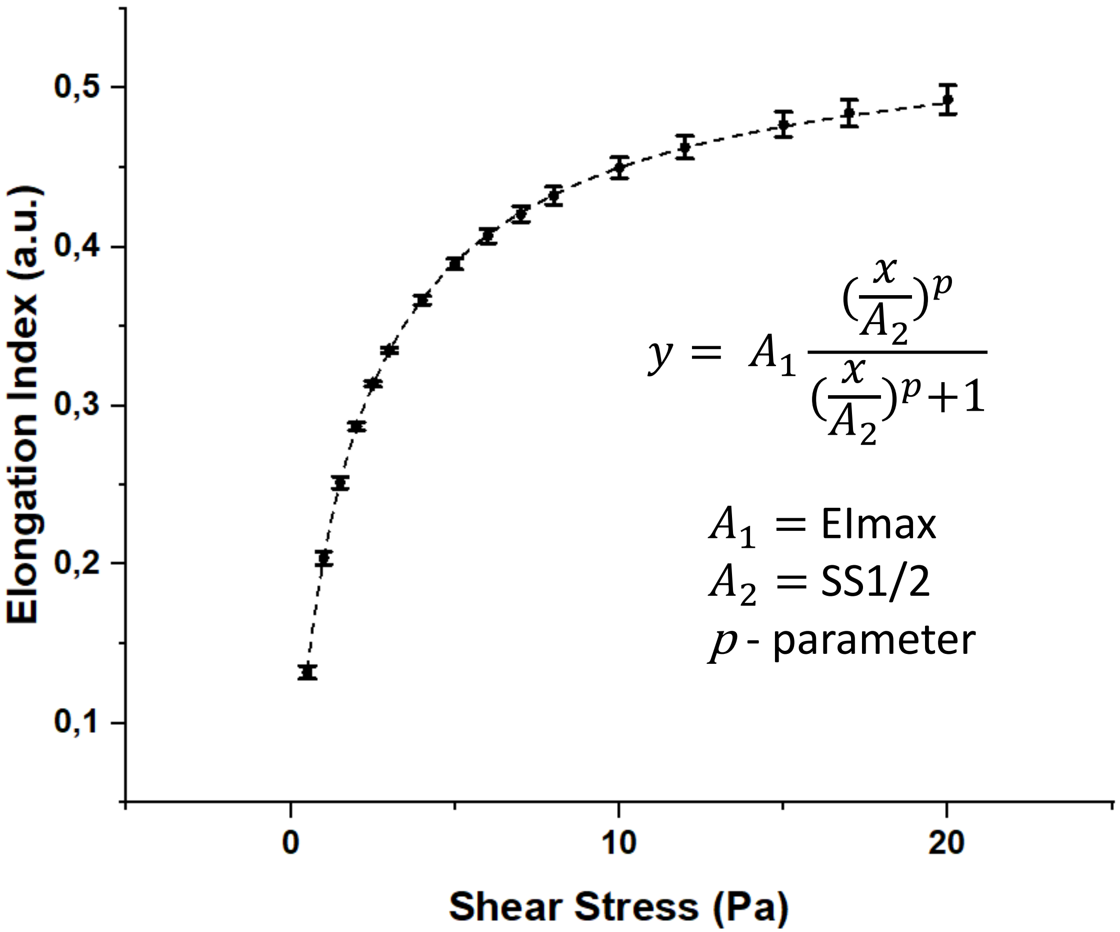

The description of the analysis of the experimental data on RBC-D changes to obtain elongation index at infinite shear stress (EImax, a.u.) and shear stress at half of this magnitude (SS1/2, Pa). In this figure we demonstrate the approximation formula and the example of the fitting. The approximation approach is described in [Baskurt, et al. Scandinavian Journal of Clinical & Laboratory Investigation, vol. 69 (7), pp. 777–788 (2009)].

5. Elongation index at infinite shear stress (EI max).

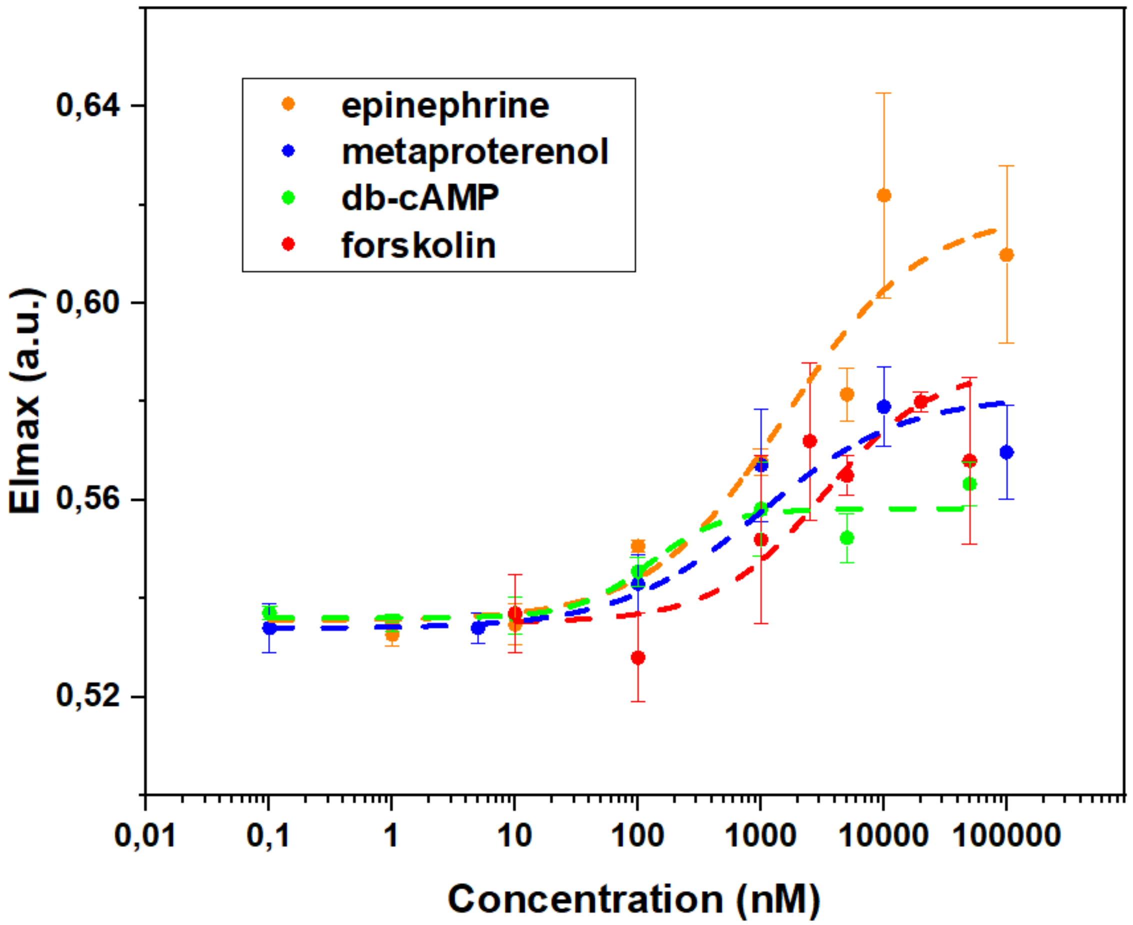

Stimulation agent	EC50
epinephrine	1576,5 ± 141,4 nM
metaproterenol	1098,8 ± 227,0 nM
db-cAMP	122,8 ± 38,7 nM
forskolin	3216,8 ± 204,1 nM

The dependence of EImax on the concentration of each stimulator of the AC-cascade. Approximation with sigmoidal function is demonstrated for each stimulator. Number of donors N=10. Error bars demonstrate the errors of the approximation performed in Origin 2018 software. EC50 values correlate by the order of magnitude with values obtained from the direct analysis of deformability curves (please refer to the fig. 3 in the text).

6. Shear stress at half of infinite elongation (SS1/2).

- The dependence of SS1/2 values on the concentration of AC-cascade stimulator. Number of donors N=10. Error bars demonstrate the errors of the approximation performed in Origin 2018 software.
- The behavior of SS1/2 values in case of epinephrine and forskolin correlates with the data obtained from deformability curves.
- The absence of significant changes of SS1/2 for db-cAMP and metaproterenol can be explained by threshold in shear stress to observe the changes in RBC-D (please refer to the fig. 2(B,C) in the text).

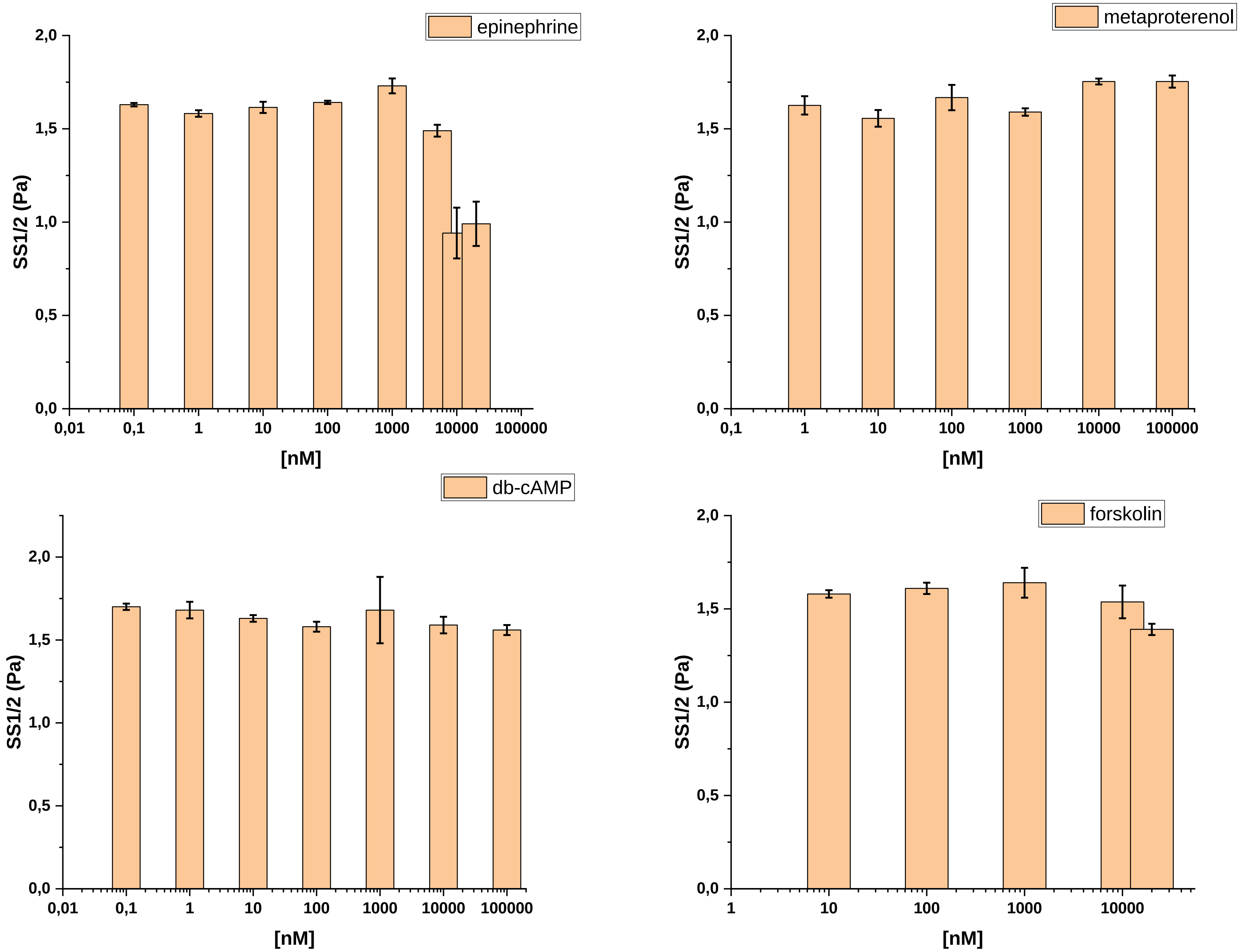

7. Microphotographs of experimental RBC suspensions under two glass slides demonstrating absence of significant morphological changes of cells at the highest concentrations of the substances. Levenhuk C510 NG; air objective 40x (0.65 N.A.).

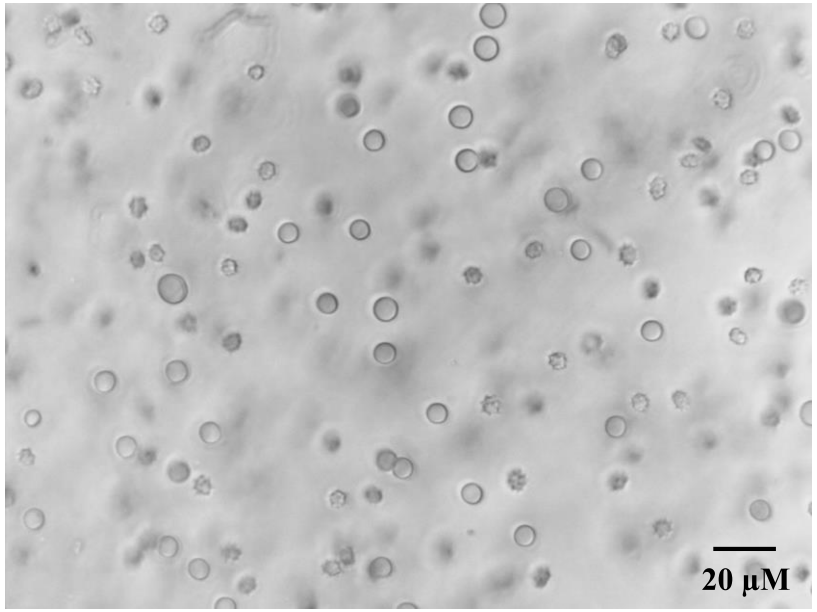

[epinephrine] 100 μM

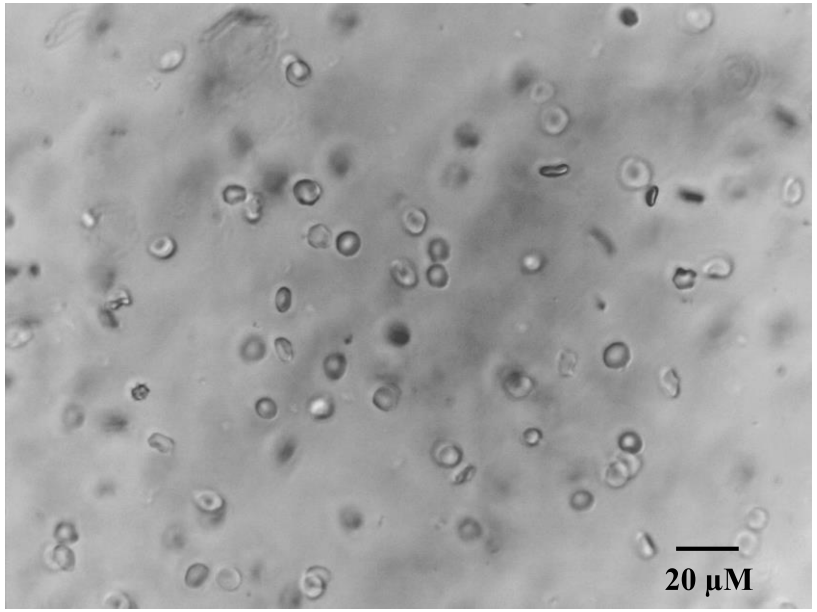

[db-cAMP] 50 μM

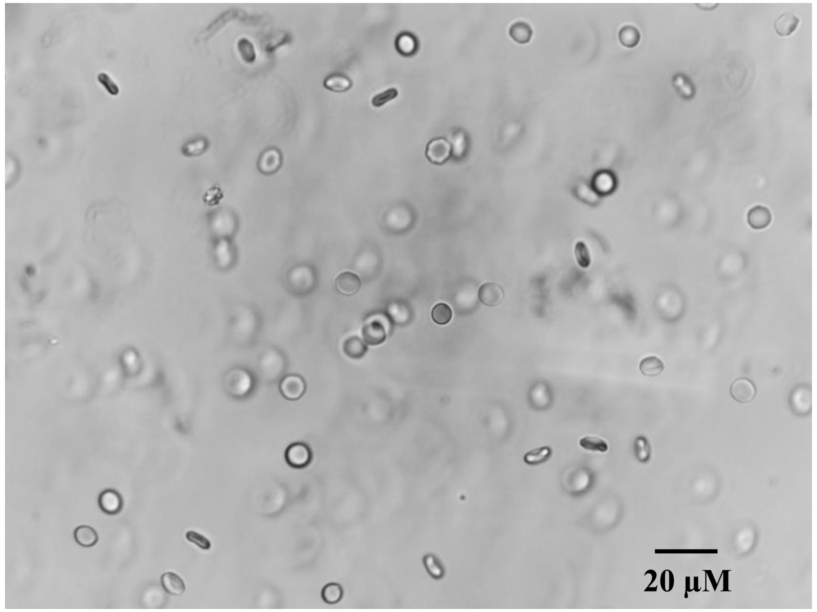

Intact

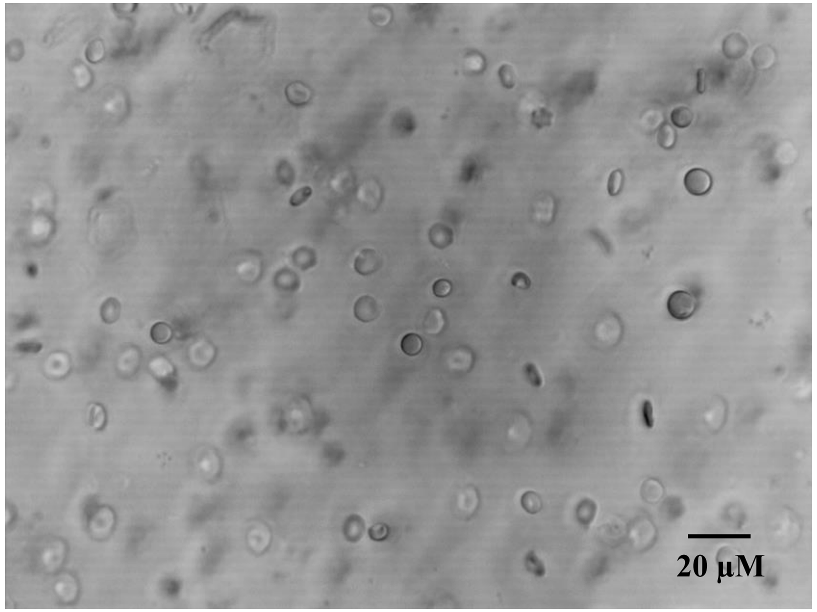

[metaproterenol] 100 μM

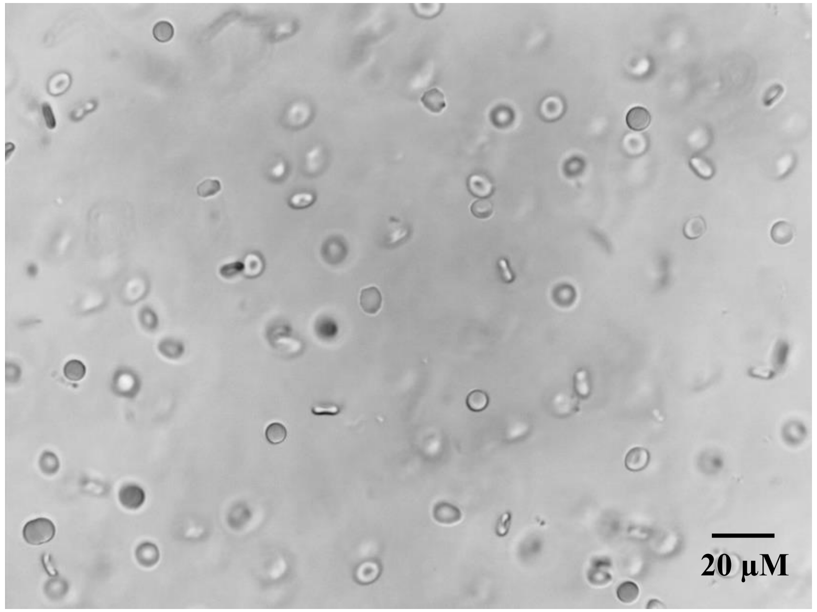

[forskolin] 60 μM

8. Adenylyl cyclase stimulation negative control.

- To provide negative control we studied the effects of bisoprolol (selective β1-adrenoreceptor antagonist capable of blocking cAMP synthesis pathway [Galandrin S. and Bouvier M., Molecular Pharmacology, vol. 70(5), pp.1575-1584 (2006)]) on RBC-D under AC-cascade stimulation (in our particular case we used catecholamine stimulation with epinephrine).

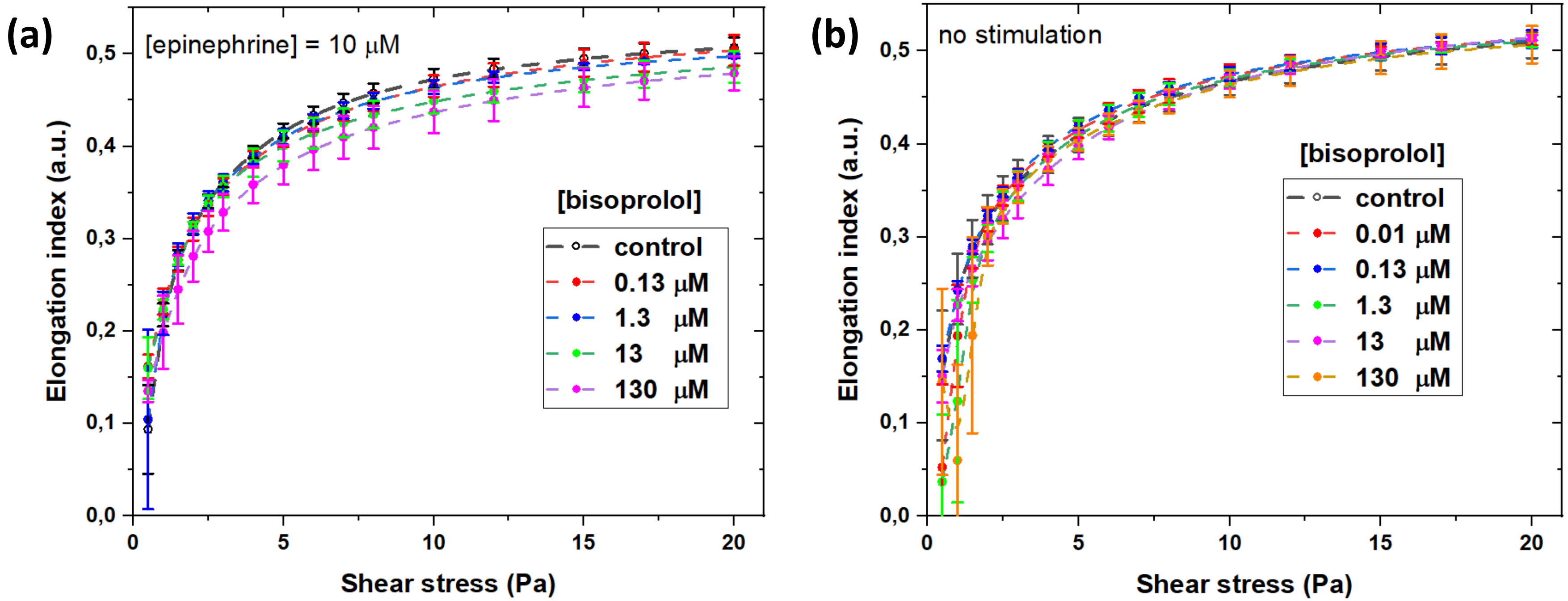

SI fig. 8. The effect of bisoprolol (inhibitor of cAMP synthesis pathway) on RBC-D:
(a) under catecholamine (epinephrine)-induced adenylyl cyclase cascade stimulation; (b) without stimulation.
Number of donors N = 5, mean values ±SD.

- After initial stimulation with epinephrine (i.e. when AC-cascade is activated and RBC-D increased) bisoprolol clearly led to the decrease in RBC-D (SI8 fig. 8(a)). When no AC-cascade stimulator added (SI fig. 8(b)) the effect on RBC-D was not significantly pronounced in comparison with control sample of intact RBC.

9. The role of anti-coagulant in the studying of adenylyl cyclase stimulation: effects of forskolin on RBC-D at different types of blood stabilization.

- The effect of forskolin on RBC-D at different type of stabilization of blood.
- Number of donors N=10, mean values ± SD.
- ‘NO ANTICOAGULANT ADDED’ sample included collecting the blood directly to the isotonic PBS containing [CaCl2] = 1 mM right after the finger pricking.
- After the sampling RBC were washed 3 times (3 min, 2500 g) in isotonic PBS at the room temperature.
- The increase in RBC-D at presence of forskolin (10 μM) was observed in all cases regardless of anticoagulant usage.

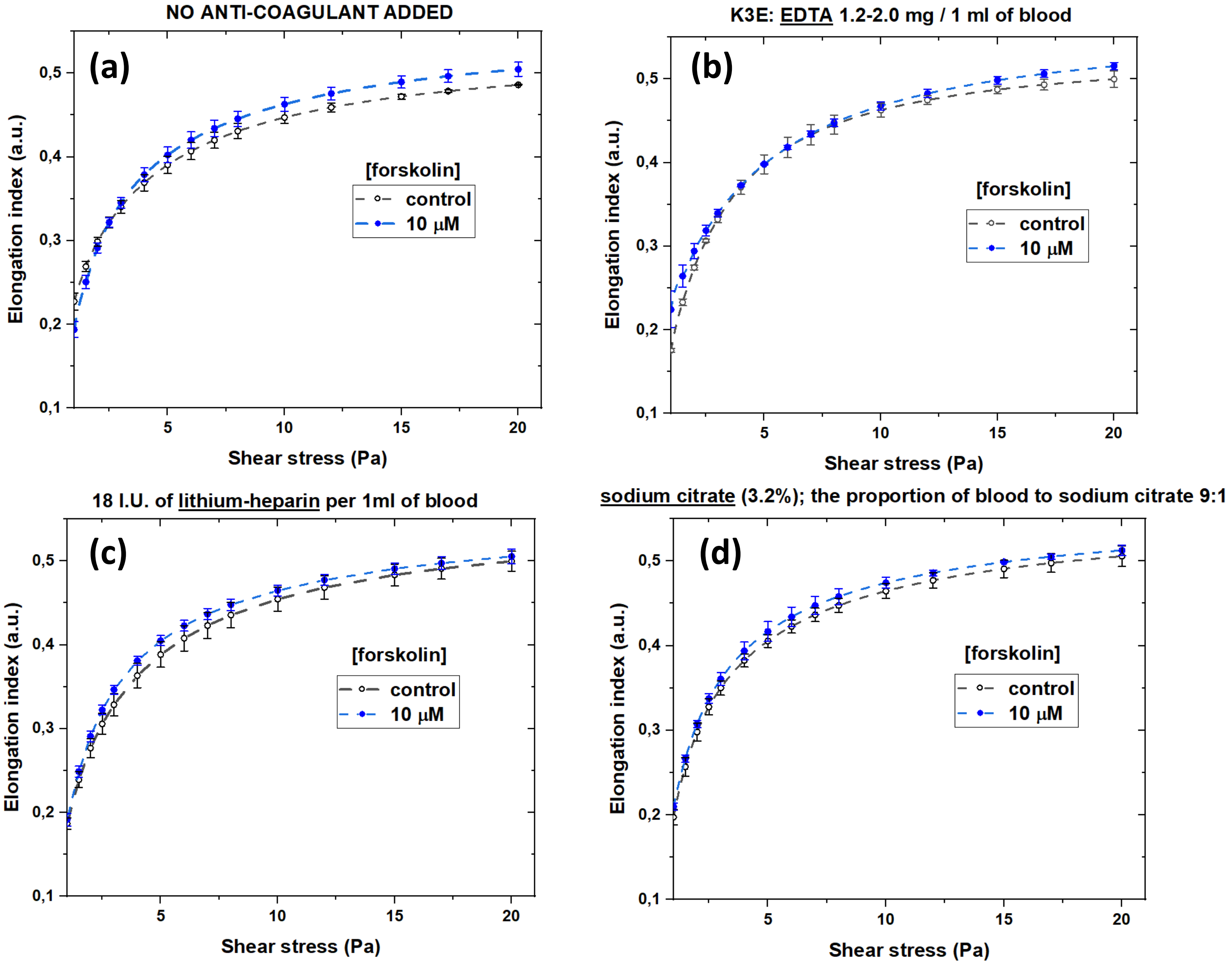

10. Single cell analysis to support the findings of adenylyl cyclase stimulation impact on RBC deformability: A study by means of optical (laser) trapping. Methodology.

- Measurements of RBC-D changes on the single-cell level were performed using optical tweezers to support the findings of the role of adenylyl cyclase stimulation in RBC microrheology regulation.

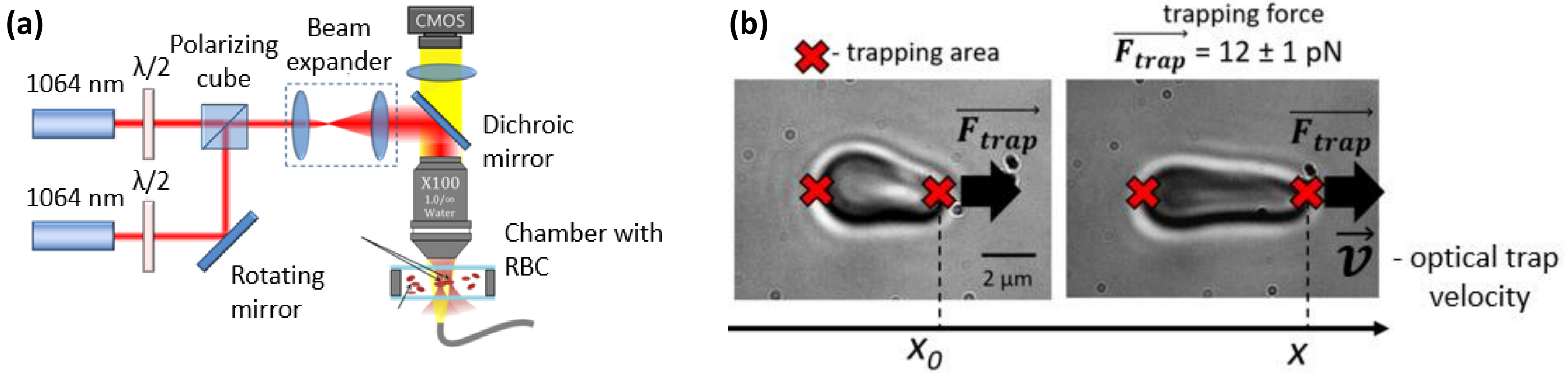

SI fig. 10. A study of effects of adenylyl cyclase cascade stimulation on the deformability of a single RBC using optical (laser) tweezers:
(a) schematic lay-out of the custom-made 2-channel optical trapping system, which allows to trap and manipulate single RBC;
(b) step-by-step methodology of the experiment:
(1) the RBC is trapped in two points (red crosses) with the trapping force Ftrap = 12±1 pN;
(2) the RBC is elongated at different velocities of the laser trap displacement;
(3) maximum elongation $\Delta x = x - x_0$ (in pixels) at a certain trap velocity is assessed as a magnitude of RBC mechanical response.

10. Single cell analysis to support the findings of adenylyl cyclase stimulation impact on RBC deformability: A study by means of optical (laser) trapping. Results.

- Low power (<10 mW) 1064 nm lasers were used to avoid the phototoxicity of the laser trapping.

- Beads in these particular experiments were not used as they may alter the RBC-D at adenylyl cyclase stimulation.

- The measurements were performed in isotonic PBS in the chamber between two glass slides covered with 1% PBS-solution of albumin.

- When epinephrine [10 μm] or db-cAMP [1 μm] were presented in the solution, the RBC were elongated easier in comparison with the intact RBC (SI fig. 11): at the presence of the stimulator at the same velocity of the laser trap, the RBC elongation (in pixels) was higher.

- The effect of epinephrine was more significant than db-cAMP, and the dependence of RBC elongation on the optical trap velocity was non-linear, which corresponds with ektacytometry results.

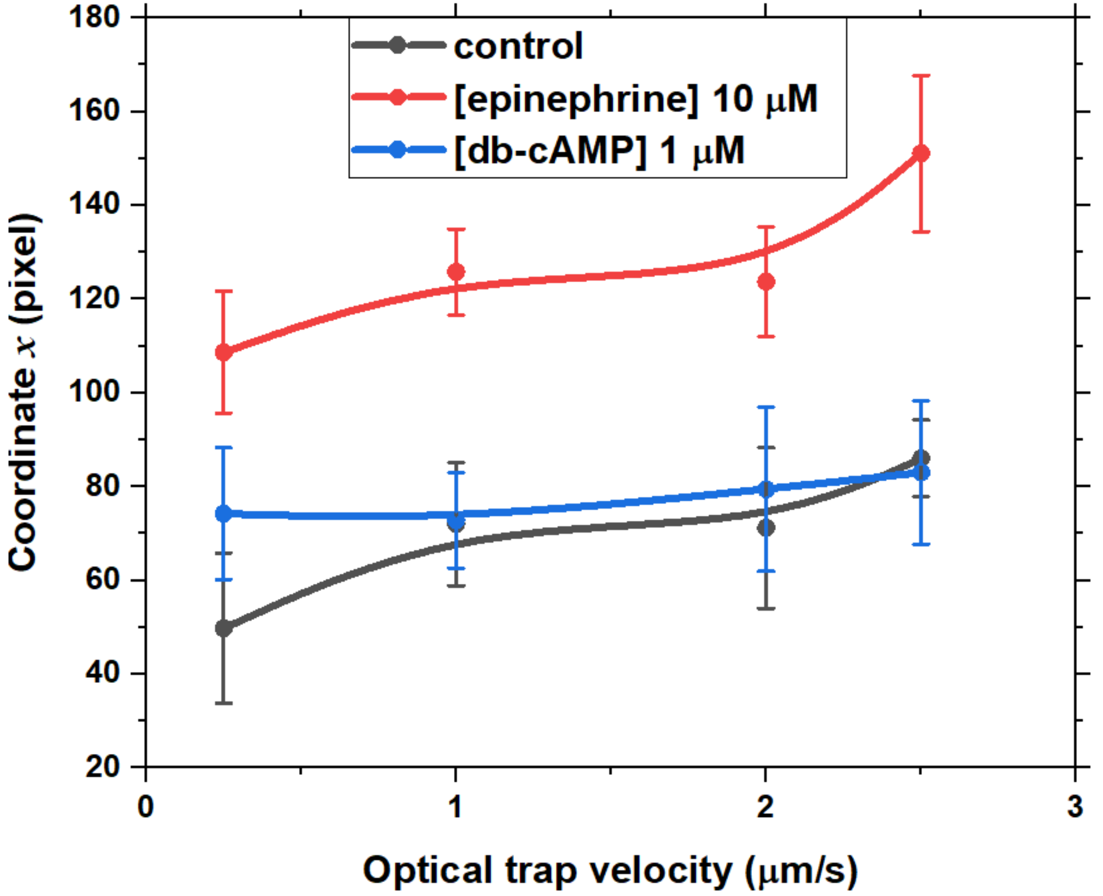

SI fig 11. Results of measuring RBC-D by the assessment of cells elongation with Ftrap = 12±1 pN at different velocity of the optical trap displacement. N=10 different RBC for each velocity. Demonstrated mean values with std. dev. as error bars.
